# Address

**Published:** 1875-04

**Authors:** H. A. Smith


### ADDRESS.

BY H. A. SMITH. D. D. S.

*Read upon retiring from the Presidential Chair at the Ohio Dental Society, December* 5, 1874.

The separation of the practice of dentistry into the operative or surgical and the so-called mechanical department, has for a long time been accepted by the profession as a most natural division. These two departments usually practiced together have formed a system, which until recently was in harmony.

In the physical body or system the first condition of complete health is, "That each organ performs its function unconsciously, nnheeded." But let an organ announce its separate existence, then already is derangement there. So it may be said of the practice of dentistry. Each of the departments performed its function in harmony and with out interruption. Now that which formerly seemed a whole, begins to show signs of wrong working; and one of the parts inclines to announce its separate existence, discord proclaims itself, and we no longer have the perfection of bodily well being.

Whence arises this discord? Why do the parts no longer adjust themselves? Nearly always when this condition, which, with our present light may be denominated as abnormal is spoken of, its cause is attributed to the decline of mechanical dentistry as an art. It is true, no doubt, that the introduction and almost universal use of the cheaper bases for artificial teeth, and the consequent influx to the ranks of the profession of a large number of persons, who, regarded simply as artisans, are posessed only of a secondary order of skill, has tended to create a feeling that the mechanical branch should not have a place, or be put on an equal footing professionally considered with the operative or surgical department. But may not the growth of this feeling also be traced largely to the fact that true dental science, which looks to the preservation of the dental organs rather than to their replacement, has greatly developed and improved? Whilst the one has passed on to a higher plane the other department has actually deteriorated. Thus the separation has become wider and wider, until dental mechanics, which is really the foster mother of operative dentistry, is in danger. The child having grown so big, is likely to crush the life out of the parent.

A discussion of the present status of mechanical dentistry involves the consideration of its claims to rank as of a profession. Indeed the whole question of the relation of dentistry to the profession of medicine might well be brought into review in this connection. But such is not my purpose. And just here I would prefer to say that I am well aware that for any one to offer any strictures upon the methods of mechanical dentistry of this day, is to incur the suspicion of an inclination to depreciate this particular branch of dentistry. But the fact that I was severely trained in my student days in what may be termed old fashioned dental mechanics, and have since spent many hours of hard labor in the dental laboratory, with results which were, I trust, creditable, in some degree, should relieve me from any such suspicion. On the contrary what I have to say on this subject has been suggested by considerations for the greatest good of the public and my fellow practitioners. That this branch of our calling is not worthy of the greatest respect, no one will dare to assert. And in fact, I doubt if there is any department in the mechanic arts, which is capable of bestowing a larger measure of positive good upon a suffering race than this, when rightly exercised. The fact that there are quite a number who largely give their attention to this department of practice, who take rank and compare favorably in point of culture and attainments with the best in the profession, is sufficient to stamp it as an honorable occupation. As a type of this class I might name Dr. John Allen, whom you all know, not only as an untiring advocate of artistic mechanical dentistry; but as well, his zeal in enforcing the obligations we are under to practice eonservative dentistry. He also goes a step further, and urges both in season and out of season, the importance of a regimen whereby a better development of the dental organ may be secured in the young. Such men as he, though devoting their time and talents to artificial dentistry would do honor to any department of medicine or surgery; and are fully competent, because of their intimate knowledge of the general science of dentistry to advise their patients as to the proper care and treatment of their teeth, and if such treatment does not come within their province as specialists they have the manhood to refer these persons to some one who is competent to perform the needful operations.

If the artificial branch of dentistry was in the hands of such as these, there would be no occasion for bringing this subject before you to-day. Then only the legitimate demand for artificial substitutes would be supplied. Instead of this however we find dental mechanics largely controlled by those who are not satisfied to supply the actual needs for artificial teeth, but really seek to create a demand for their wares by assuming to be competent to advise their patients as to the requirements of their case, with the result very frequently that their teeth are criminally sacrificed.

Now I think that all must admit that it is impossible for any one trained as many of these so-called dentists are in the laboratory only, to practice the higher departments of dentistry. And I mean by the higher departments, that knowledge and skill whereby we are enabled in a large proportion of cases, when placed entirely under our control to conserve the natural teeth, It includes also the ability to recognise and treat properly, the variety of abnormal conditions that come strictly within the province of dentistry; as well as a knowledge of that particular branch of dental science hitherto so sadly neglected—dental hygiene. It has justly been said, the time will soon come when it will be regarded as the greatest achievement of the medical art, not that this man or that is cured when he is sick, but when it prevents a whole community from falling ill. And of the dental practitioner it will in time also be said, when he can, by advice or treatment, prevent disease of the dental organs, that he has achieved the highest success in the dental art.

There are great responsibilities which must of necessity attend to fit him to assume these duties and responsibilities. Each tach to the calling of a dentist, and the student who desires to qualify himself for the serious duties of his profession, can not afford to divide his time in any work which does not directly year there is a striving after a higher professional standard and this can only be met with a more thorough training of those who are about to enter upon this pursuit. Nothing short of a general knowledge of the human organism, and the modes and conditions of its action in health. A special knowledge of the structure and function of the organs with which he has most to do; their relations to other organs; the the diseases to which they are liable; and the best means of restoring them to health and usefulness will rightly qualify the dentist for his profession Superadded to all this he must possess manipulative ability. He must be educated in his finger's end, before he can put in practice his varied acquirements.

Granting then that the dental practitioner ought to be a person who has pursued this range of study, is there anything in the domain of mechanical dentistry which calls for, or would stimulate one to strive for a mastery of the subjects I have named? It is true that the study of anatomy, especially of the facial regions, and perhaps physiology to a limited extent, may be regarded as a requisite in dental mechanics. But these would be pursued with a purpose similar to that which would influence the artist to undertake the study of these sciences. The mechanical dentist, imbued as he is with the practical spirit of the times, would regard all this study and training as a waste of effort.

The dental profession is covetous of a recognition as a part of the medical fraternity. But can we ever hope for such recognition while he who is simply able to construct a set of artificial teeth is regarded by the public and treated by ourselves as a qualified dental practitioner? And this leads us to consider the separation of these two branches of dental practice. A friend who is an experienced and accomplished dentist, recently said to me, that he regarded the agitation of this question of separation as premature, that it was slowly being effected, and in due season, the public would very generally discriminate between the operative and mechanical man, according to their need of either. And thus imperceptibly almost, and without any jarring or conflict of interests each branch of dentistry would take its proper place either as of a professional or a mechanical art.

All this may be true, bnt whether we will or no, this very question is now prominently before us. It has for a considerable time occupied the attention of thoughtful men in the profession, and has been more or less discussed in a quiet way amongst them. More lately however a few have been emboldened to take a decided stand and publicly urge the necessity of each department being pursued as a distinct calling. Notably Dr. Allport in his very able address recently delivered before the American Academy of Dental Science, urges in a forceable manner the need of such a separation. He says,—

"The yoking together of the two callings seemed to be a necessity of the then condition of the practice, at the time they were joined, and has resulted in great good. But the development of the practice has now brought us to a point where it is clear a new departure should be taken, the copartnership dissolved and each department followed as a distinct and separate calling; both no longer in our private offices or in our colleges be taught as *one;* and the term dentist dropped from our nomenclature."

Dr. Allport puts the subject fairly before the profession in the most radical form—even to proposing the adoption of new names for each of the departments. And since your Executive Committee proposes this very question for discussion at the present meeting it would seem that its agitation had already commenced. The consideration of this whole subject, in the order suggested, I have no doubt will prove profitable to all of us, if you will but give it that thoughtful attention which, as an intelligent body of practical dentists, you are capable of.

There are some difficulties in the way of this separation—especially outside of our cities and the more populous towns, that will be readily suggested to you, which it would be well for you to carefully examine, that they may not be given more weight than they deserve. Some of these objections have, all along through the history of medicine, been opposed to the separation of the practice into specialties. The divisions in practical medicine and surgery have steadily gone on, however, until we are threatened with a specialty for nearly every important organ of the human system. A reflex action will occur in due season, no doubt, and the practice of medicine and surgery will be separated into a limited number of departments that will be indicated as founded on a certain natural basis.

With our present knowledge of the subject, let us suppose for a moment that the diseases and defects of the dental organs were treated by the general surgeon, would not the fact that for the successful treatment and needed operations on the teeth a high degree of special training is requisite, clearly indicate the need of placing this department in the hands of specialists? For the same reasons the surgical treatment of the eye is very generally given over to the ophthalmologist.

If, then, it can be shown that there is quite enough in medical and surgical, or operative dentistry, to require the undivided attention of both the student and practitioner, to the exclusion of mechanical dentistry, should not this afford sufficient reason or basis for their separate practice?

As sometimes sung:

> "If a man could be sure
> That his life would endure
> For the space of a hundred long years."

He might, if a dentist, do much to perfect himself in the whole range of the science and art of his profession, which now, in the hurry and press of this mercenary age, seems impracticable. Superficial knowledge, the result of imperfect training, is a marked defect in the practice of the arts and sciences as well as the trades of the day. And we can truthfully say that dentistry is in no sense an exception to the rule. And I put it to you, that unless it can be shown that the teaching of mechanical dentistry to our students will be an assistance or aid to qualify them for the practice of legitimate dentistry, it must be admitted to be a waste of time—if indeed it does not actually lead them away from the one central idea or thought which must ever animate those who expect to fill the truc sphere of a dentist, viz: The importance of preserving the natural teeth. If the student or practitioner divides his attention with a pursuit that is practicably based upon our inability to accomplish this end, he soon comes to distrust himself and his abilities in the direction of conservative dentistry. And very soon his patrons partake of this weakening. Then follows the feeble apology that his patients do not want their teeth saved. And it is in this manner that whole communities are demoralized, as to a proper appreciation of conservative dentistry.

But no matter how desirable this separation may be, both for the welfare of the community and the elevation and appreciation of the profession, it can not be accomplished by any short-eut. No manifesto or resolution adopted by this Society would tend to hasten such a consummation. It is clearly within your province, however, I think, to discuss this subject, after the mature reflection which you have paid it, no doubt, and give expression to your individual opinions, unbiased and as free from that prejudice which self-interest and fondness for an old custom or practice is apt to engender.

This Soeiety has frequently been commended for the zeal which its membership has ever shown, since its first organization, to assist in raising the standard of qualifications requisite for admission to the profession. That you originated—though somewhat as an experiment—and have maintained a law to regulate the practice of dentistry in the State, whereby a large number have been subjected to examination and required to show that they were possessed of attainments equal at least to those of the average legitimate practitioner is a declaration that you were not satisfied with the old order of things. And in the effort that is now being vigorously made to put dentistry abreast with the recognized specialties of the medical art, it is expected that you may be found on the side and ready to recommend or adopt measures that seem wise and practicable, to speed the day when dentistry shall no longer be stigmatized as simply a mechanical art.

And though it may not be expected of those of you who are established in practice and bound down to old customs, either from habit or necessity, that you will give up legitimate business in either departments of dentistry, and at once become specialists; it will be no less your duty if you are satisfied that for the mutual interests of the profession and those who seek their services, that the practice should be divided to duly impress your conviction upon any who may be just entering upon the profession. A word spoken in season may lead the student to concentrate his energies in the direction most in accord with his tastes, and thus develop in himself the highest degree of proficiency possible to him.

A reform such as I have indicated can, in this way, be inaugurated, maintained and so speedily accomplished that some now present will wonder and ask why it was not always so.

It is not profitable, however, to indulge over much in anticipation of the future.

There are the questions of the hour. Subjects that present themselves and bear upon our everyday's duty. All these must receive the greater share of our attention. Carlyle has eloquently said:

"It is no very good symptom either of nations or individuals, that they deal much in ratiocination. Happy men are full of the present, for its bounty suffices them; and wise men, also, for its duties engage them. Our grand business undoubtedly is, not to *see* what lies dimly at a distance, but to *do* what lies clearly at hand.

"Know'st thou *Yesterday*, its aim and reason?
Work'st thou well *To-day* for worthy things?
Then calmly wait the *Morrow's* hidden season,
And fear not thou, what hap so e'er it brings."

